# The role of serum thymidine kinase 1 activity in neoadjuvant-treated HER2-positive breast cancer: biomarker analysis from the Swedish phase II randomized PREDIX HER2 trial

**DOI:** 10.1007/s10549-023-07200-x

**Published:** 2024-01-04

**Authors:** Yajing Zhu, Ioannis Zerdes, Alexios Matikas, Ivette Raices Cruz, Mattias Bergqvist, Ellinor Elinder, Ana Bosch, Henrik Lindman, Zakaria Einbeigi, Anne Andersson, Lena Carlsson, Ann Charlotte Dreifaldt, Erika Isaksson-Friman, Mats Hellstrom, Hemming Johansson, Kang Wang, Jonas C. S. Bergh, Thomas Hatschek, Theodoros Foukakis

**Affiliations:** 1 Department of Oncology-Pathology, Karolinska Institutet, Karolinska Vägen A2:07, Solna, 171 64 Stockholm, Sweden; 2 Breast Center, Theme Cancer, Karolinska University Hospital, Stockholm, Sweden; 3 Division of Biostatistics, Institute of Environmental Medicine, Karolinska Institutet, Stockholm, Sweden; 4 Biovica International, Uppsala Science Park, Uppsala, Sweden; 5 Department of Oncology, South Hospital, Stockholm, Sweden; 6 Department of Hematology, Oncology and Radiation Physics, Skåne University Hospital, Lund, Sweden; 7 Department of Oncology, Uppsala University Hospital, Uppsala, Sweden; 8 Department of Oncology, Southern Älvsborg Hospital, Borås, Sweden; 9 Umeå University, Umeå, Sweden; 10 Department of Oncology, Sundsvall Hospital, Sundsvall, Sweden; 11 Department of Oncology, Örebro University Hospital, Örebro, Sweden; 12 Department of Oncology, St Göran Hospital, Stockholm, Sweden; 13 Centre for Clinical Cancer Studies, Theme Cancer, Karolinska University Hospital, Stockholm, Sweden

**Keywords:** Thymidine kinase, HER2 + breast cancer, Biomarker, Neoadjuvant treatment, Prognosis

## Abstract

**Background:**

Thymidine kinase 1 (TK1) plays a pivotal role in DNA synthesis and cellular proliferation. TK1 has been studied as a prognostic marker and as an early indicator of treatment response in human epidermal growth factor 2 (HER2)-negative early and metastatic breast cancer (BC). However, the prognostic and predictive value of serial TK1 activity in HER2-positive BC remains unknown.

**Methods:**

In the PREDIX HER2 trial, 197 HER2-positive BC patients were randomized to neoadjuvant trastuzumab, pertuzumab, and docetaxel (DPH) or trastuzumab emtansine (T-DM1), followed by surgery and adjuvant epirubicin and cyclophosphamide. Serum samples were prospectively collected from all participants at multiple timepoints: at baseline, after cycle 1, 2, 4, and 6, at end of adjuvant therapy, annually for a total period of 5 years and/or at the time of recurrence. The associations of sTK1 activity with baseline characteristics, pathologic complete response (pCR), event-free survival (EFS), and disease-free survival (DFS) were evaluated.

**Results:**

No association was detected between baseline sTK1 levels and all the baseline clinicopathologic characteristics. An increase of TK1 activity from baseline to cycle 2 was seen in all cases. sTK1 level at baseline, after 2 and 4 cycles was not associated with pCR status. After a median follow-up of 58 months, 23 patients had EFS events. There was no significant effect between baseline or cycle 2 sTK1 activity and time to event. A non-significant trend was noted among patents with residual disease (non-pCR) and high sTK1 activity at the end of treatment visit, indicating a potentially worse long-term prognosis.

**Conclusion:**

sTK1 activity increased following neoadjuvant therapy for HER2-positive BC but was not associated with patient outcomes or treatment benefit. However, the post-surgery prognostic value in patients that have not attained pCR warrants further investigation.

**Trial registration:**

ClinicalTrials.gov, NCT02568839. Registered on 6 October 2015.

**Supplementary Information:**

The online version contains supplementary material available at 10.1007/s10549-023-07200-x.

## Introduction

Uncontrolled cell proliferation is a key hallmark of cancer [1]. Thymidine Kinase 1(TK1), a strictly cell cycle-regulated enzyme and well-characterized proliferation marker, is essential during DNA precursor synthesis. TK1 levels and activity are low or undetectable in resting cells but increase significantly from late G1 to late S-phase in proliferating cells [2]. The role of cellular TK1 as a potential biomarker has been previously evaluated in breast and other cancer types, mostly associated with worse prognosis [3, 4]. The advantage of minimally invasive measurement of TK1 activity in serum samples enables its serial evaluation during different disease phases-as compared to tissue-based markers such as mitotic count and Ki-67, while its reliable and reproducible quantification has been validated in several large prospective cohorts [5–7].

The USA Food and Drug Administration approved the use of sTK1 activity in 2022 as a biomarker for monitoring disease progression in previously diagnosed hormone receptor-positive (HR +), HER2-negative metastatic postmenopausal breast cancer(mBC) patients based on the results of the SWOG S0226 trial[7] and subsequently validated in a recent prospective trial of 287 mBC patients receiving first-line CDK4/6 inhibitor(CDK4/6i) in combination with endocrine therapy (ET) [8]. Its impact on physician’s decision making on HR + mBC is currently under evaluation in another prospective trial[9]. In early BC patients, effective neoadjuvant treatment is becoming the standard of care, but the identification and validation of potential biomarkers of early response remains an unmet need. A recent study demonstrated the utility of serum TK1 activity for monitoring responses to neoadjuvant CDK4/6i in early HR + BC patients[10]. In addition, we have previously shown that serial measurement of serum TK1 activity during neoadjuvant chemotherapy (NACT) might provide long-term prognostic information[11]. However, there is currently limited data regarding the utility of TK1 as a predictive or prognostic marker in HER2-positive BC.

In the phase II randomized PREDIX HER2 trial, we previously reported that the efficacy of standard neoadjuvant combination of trastuzumab, pertuzumab, and docetaxel(DPH) treatment was not superior to trastuzumab emtansine(T-DM1) in terms of pathologic complete response (pCR) and long-term survival, while patients treated with T-DM1 had a markedly lower frequency of adverse effects and significantly better quality of life during the neoadjuvant period [12, 13]. In this study, we evaluated the potential predictive and prognostic value of baseline and serial levels of serum TK1 in patients with HER2 + early BC enrolled in the PREDIX HER2 trial.

## Methods

### Clinical trial, endpoints, and sample collection

PREDIX HER2 is a phase II, randomized, multicenter, academic clinical trial, conducted between December 2014 and October 2018 in nine centers across Sweden (ClinicalTrials.gov identifier NCT02568839). The study enrolled male or female patients aged 18 years or older with ERBB2-positive tumors larger than 20 mm and/or verified lymph node metastases. Patients were randomized in a 1:1 ratio to receive either six courses of docetaxel (first dose, 75 mg/m^2^, then 100 mg/m^2^), subcutaneous trastuzumab (600 mg), and pertuzumab (loading dose, 840 mg, then 420 mg), or six courses of T-DM1 (3.6 mg/kg).

The primary endpoint of the study was objective pathologic response, with pathologic complete response (pCR) defined as the absence of invasive tumor in the breast and lymph nodes (ypT0/Tis, ypN0). Event-free survival (EFS) was defined as the time from the date of randomization to the occurrence of the first event, including progression during treatment, locoregional or distant recurrence, contralateral breast cancer, other malignancy, or death from any cause. Disease-free survival (DFS) was defined as the time from the date of surgery to the first appearance of locoregional or distant recurrence, contralateral breast cancer, any cancer from other primary sites, or death from any cause.

**Fig. 1:**
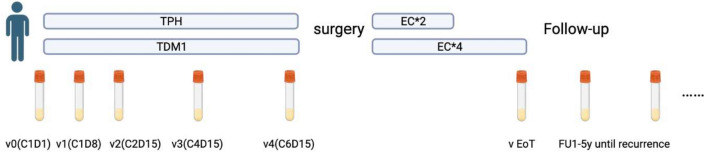
Study schema for the PREDIX HER2 trial. Serial and blood collections occurred at at visit 0(C1D1), visit 1(C1D8), visit 2(C2D15), visit3(C4D15), visit 4(C8D15), visit EoT(end of treatment), visit FU1-5y(yearly follow-up), visit R(time of recurrence) This figure was created by BioRender.com (BioRender, Toronto, ON, Canada)

As shown in Fig. 1, blood samples were collected from all patients at baseline (visit 0), 16 ± 2 days after 2 cycles of treatment (visit 2), 16 ± 2 days after cycle 4 (visit 3), 16 ± 2 days after cycle 6 (visit 4), at the time of adjuvant treatment ends (visit EoT) and, where applicable, at the time of recurrence (visit R). Due to a protocol amendment, blood at visit 1(8 ± 2 days after cycle 1) was only collected from some of the patients treated at Karolinska University Hospital. This correlative analysis is reported in accordance with the REMARK criteria (REporting recommendations for tumor MARKer, supplementary Table 1).

### Measurement of TK1 activity

The study employed the ELISA-based DiviTum® TKa assay (Biovica, Sweden) to determine the enzymatic activity of sTK1 in serum samples. The assay was performed on two aliquots of approximately 1 mL serum for each timepoint, following the manufacturer's instructions and as previously described [11]. Briefly, the serum samples were mixed with a reaction buffer and incubated with a 96-well microtiter plate. The TK reaction phosphorylated bromodeoxyuridine (BrdU), a thymidine analogue, to BrdU-monophosphate, which was further phosphorylated into BrdU triphosphate. An anti-BrdU monoclonal antibody conjugated to enzyme alkaline phosphatase and a chromogenic substrate were used to detect BrdU triphosphate, resulting in the production of a yellow reaction product. The enzymatic activity of TK1 was expressed as DiviTum® unit of Activity (DuA), which were calculated using known TK activity values from reference sample of recombinant TK within a measuring range of 45 to 3081 DuA. All samples were analyzed at Biovica laboratories in Uppsala, Sweden, blinded to patient and tumor characteristics.

### Statistical analysis

**Fig. 2:**
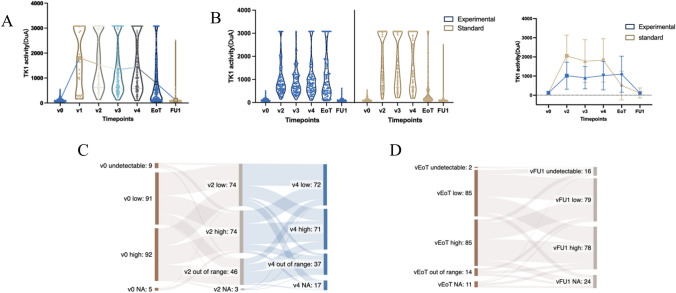
**A** median sTK1 change over time for all patients and **B** by treatment arm **C** sTK1 activity shifts from baseline to visit 2 and visit 4 and **D** from visit EoT to visit FU1

Violin plots were generated to show serum TK1 activity by time point in all patients. Undetectable TK1 activity of < 45 DuA at baseline and extreme high TK1 activity of > 3081 DuA were regarded as 45 and 3081 in the description of TK1 levels over time (Fig. 2A and B). Line plots displayed the levels of serum TK1 activity by time point in all patients and by treatment groups. For categorical variables, the distribution of TK1 levels in standard clinicopathological subgroups was compared using Chi-square test or the exact Fisher test. For continuous variables, difference in mean or median between groups was assessed using *t*-student test or ANOVA-test (parametric) or Mann Whitney test or Kruskal Wallis (non-parametric) as appropriate.

The association of TK1 activity with pCR, EFS, and DFS was tested with univariate logistic regression and Cox regression. Multivariate analyses, including factors that were statistically significant in the univariate analyses and/or were clinically relevant, were applied to assess the adjusted odds ratios and hazard ratios. All *p* values are two-sided. All statistical analyses, descriptive and inferential, were performed with R version 4.2.1 software (R Foundation for Statistical Computing, Vienna, Austria) and GraphPad Prism (GraphPad Prism version 8.0, San Diego, CA, USA).

## Results

### Patient characteristics and outcomes

A total of 202 patients were initially enrolled in the PREDIX HER2 trial, five patients were excluded from further analysis (three patients withdrew consent and two patients received a diagnosis of disseminated disease before treatment initiation). The intention-to-treat population consisted of 197 patients (99 patients in the standard group and 98 patients in the investigational group), all were evaluable for the current analysis. Patient characteristics and treatment details have been previously described[12] and are shown in brief in Supplementary Fig. 1.

In total, 1144 specimens were successfully analyzed for serum TK1 activity at following timepoints: baseline(*n* = 192), visit 1(*n* = 31), visit 2(*n* = 194), visit 3(*n* = 181), visit 4(*n* = 180), visit EoT(*n* = 186), visit FU1(*n* = 173). Six specimens were collected at visit R. At baseline, 183 (95.3%) patients had a TK1 activity value in the detection range of 45 to 3081 DuA, 9 (4,6%) had an undetectable value, and 5 patients had missing data. The median (interquartile range [IQR]) pretreatment level of available sTK1 values was 101.4 (74.78–150.4) DuA.

Table 1 presents the distribution of patient characteristics according to baseline TK1 activity. No association was detected with respect to age, tumor grade, hormone receptor status, Ki-67 status, or TILs percentage. The median follow-up for patients with available baseline TK1 data was 58 (range, 17–88) months.

**Table 1:** Distribution of clinicopathologic characteristics of patients, according to baseline TK1 activity (median of detectable TK1 value as cut-off for high and low group)

	Total available	Baseline sTK1			*p* value
		Undetectable	Low	High	
Age					0.81^a^
≤ 59	145	8	69	68	
≥ 60	47	1	23	23	
Menopausal					0.75^a^
Premenopausal	94	3	48	43	
Perimenopausal or postmenopausal	91	6	41	44	
NA	7	0	3	4	
Treatment arm					0.46^a^
DPH	94	3	43	48	
TDM1	98	6	49	43	
Tumor size					0.75^a^
≤ 20	34	2	17	15	
21–50	121	7	55	59	
> 50	34	14	17	3	
NA	6	0	6	0	
Grade					0.22^a^
I–II	78	4	32	42	
III	91	5	49	37	
NA	23	0	11	12	
Node					0.51^a^
N0	106	4	48	54	
N +	86	5	44	37	
ER					0.17^a^
ER– and PR–	71	1	32	38	
ER + and PR– or PR +	121	8	60	53	
HER2					0.12^a^
ERBB2 2 +	38	4	15	19	
ERBB2 3 +	154	5	77	72	
Ki67					0.46^a^
< median	77	4	33	40	
≥ median	114	5	59	50	
NA	1	0	1	0	
TILs					0.59^a^
< 10	64	3	34	26	
≥ 10	106	5	49	52	
NA	23	1	9	13	
Follow-up(mon[median,[min–max])	58(17–88)	63.0(39–77)	58.5(37–88)	54.0(27–77)	0.55^c^
Number of events					
pCR events	87	2	39	46	0.2^a^
EFS events	21	2	9	10	0.44^a^
Switch-over					0.46^a^
No change	166	9	79	78	
DPH → TDM1	17	0	8	9	
TDM1 → DPH	9	0	5	4	

^a^P value was calculated from Chi-Square test

^c^P value was calculated from Anova test

### sTK1 activity kinetics during treatment

In sequential samples, the levels of sTK1 activity in all patients and by treatment arms are summarized in Supplementary Table 2. The sTK1 activity kinetics are illustrated in Fig. 2A. Generally, TK1 activity was low at baseline for most patients. It significantly increased (*p* < 0.001) after one cycle of treatment, remained relatively stable during the neoadjuvant phase, and decreased at the end of adjuvant treatment. The median level of sTK1 activity subsequently decreased to approximately the same value as at baseline at the 1-year follow-up. The fluctuation of median sTK1 activity from baseline to visit 2 was higher in DPH arm than in T-DM1 arm, the level remained at high level of above 1000 DuA during the DPH treatment and decreased at visit EoT, while sTK1 remained at the intermediate high level of around 1000 DuA during neoadjuvant phases of T-DM1 treatment and at the high level at visit EoT (Fig. 2B).

sTK1 activity level was explored as a categorical variable by dividing patients into three groups based on median value at baseline: undetectable (< 45 DuA), low (45 DuA ≤ value ≤ median), high (> median). At subsequent timepoints, patients were categorized into three groups based on the median value of sTK1 at each timepoint: low (< median), high (median ≤ value ≤ 3081 DuA), and out of range (> 3081 DuA). A significantly higher proportion of patients had out of range sTK1 activity at visit 2 and 4 in the standard than in the experimental arm, while conversely, higher proportion of patients had low sTK1 activity in the standard arm at visit EoT, and there is no difference in visit FU1(Supplementary Table 3). The dynamic group change from baseline to visit 2 and visit 4 is shown in Fig. 2C and D shows the flow of group change from visit EoT to visit FU1.

### Association between sTK1 activity levels and pCR

To evaluate sTK1 level as an early marker of therapy response, we assessed the association of sTK1 levels at baseline, visit 2 and visit 4 and their kinetics with pCR. The median (IQR) sTK1 levels over time for pCR and non-pCR cases are illustrated in line chart (Supplementary Fig. 2A). Number of patients distributed in three groups divided by median value in treatment arms are shown in Supplementary Fig. 2B. sTK1 level at baseline, visit 2 and visit 4 did not have a significant effect on pCR status in adjusted logistic regression model (Table 2).

**Table 2:** sTK1 level at baseline, visit 2 and visit 4 and its associations with pCR

	Logistic regression model			
	Unadjusted model		Adjusted model^a^	
	Estimates *β* (95% CI)	*p* value	Estimates β(95% CI)	*p* value
sTK1 at baseline	− 1.253 (− 3.157; 0.167)	0.118	− 0.624 (− 2.882; 1.338)	0.551
High vs Undetectable	1.275 (− 0.207; 3.215)	0.124	1.209 (− 0.325; 3.178)	0.156
Low vs Undetectable	0.946 (− 0.537; 2.886)	0.254	0.775 (− 0.761; 2.745)	0.363
sTK1 at visit2	− 0.272 (− 0.739; 0.185)	0.246	0.56 (− 0.724; 1.865)	0.393
High vs Low	0.005 (− 0.595; 0.706)	0.868	− 0.131 (− 0.894; 0.625)	0.734
Above range vs Low	0.098 (− 0.646; 0.839)	0.796	0.22 (− 0.735; 1.183)	0.651
sTK1 at visit4	− 0.394 (− 0.874; 0.072)	0.101	0.253 (− 1.122; 1.634)	0.717
High vs Low	0.478 (− 0.181; 1.147)	0.157	0.404 (− -0.363; 1.179)	0.303
Above range vs Low	− 0.103 (− 0.931; 0.706)	0.805	0.047 (− 0.944; 1.034)	0.926

^a^Adjusted for Ki67, treatment arm, tumor grade, tumor size, ER status and node status

### Clinical outcomes according to sTK1 level at baseline and follow-up timepoints during therapy

**Fig. 3:**
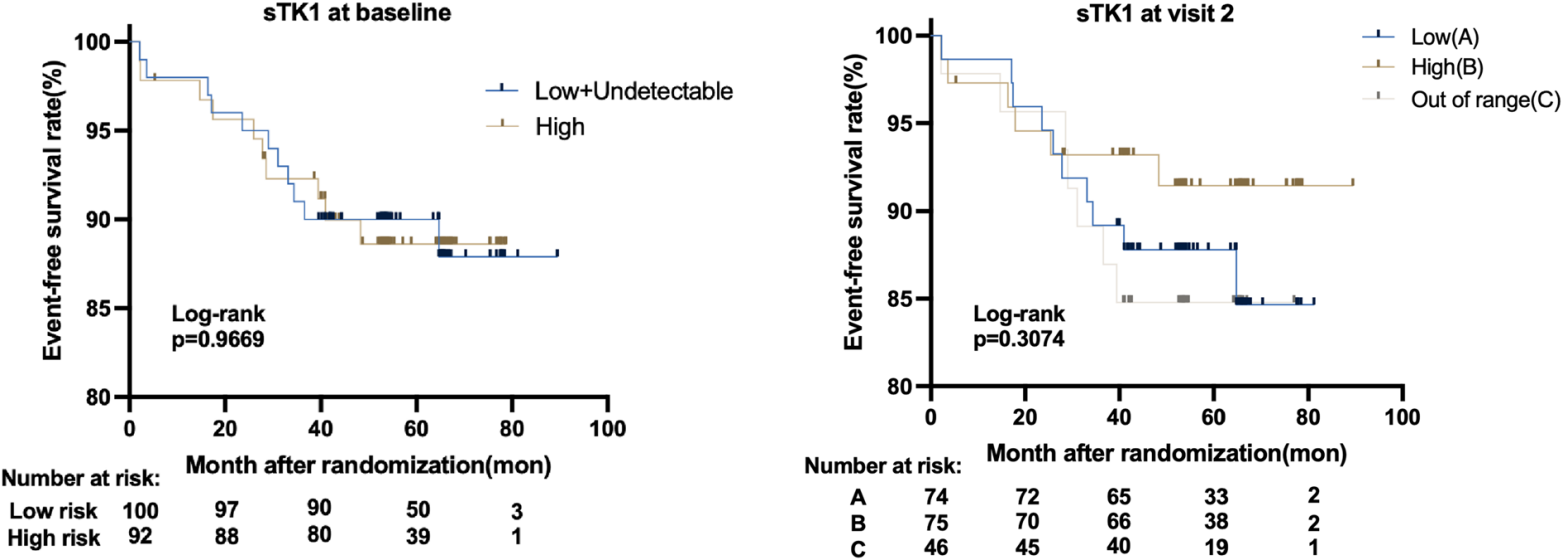
Baseline and visit 2 sTK1 and its correlations to EFS

Finally, we investigated the association between sTK1 levels and long-term prognosis. sTK1 levels at baseline and visit 2 were not associated with EFS (Fig. 3, Table 3).

**Table 3:** EFS probability according to sTK1 level at baseline and visit 2

	Cox regression model			
	Unadjusted model		Adjusted model^a^	
	HR (95% CI)	*p* value	HR (95% CI)	*p* value
sTK1 at baseline(continuous)	1.003 (0.999–1.008)	0.164	1.002 (0.996–1.007)	0.559
sTK1 at baseline				
High vs Undetectable	0.494 (0.108–2.255)	0.363	0.365 (0.07–1.894)	0.23
Low vs Undetectable	0.422 (0.091–0.955)	0.27	0.253 (0.05–0.288)	0.098
sTK1 at visit2				
High vs Low	1.09 (0.418–2.844)	0.86	1.497 (0.501–4.467)	0.47
Above range vs Low	1.28 (0.443–3.704)	0.649	2.878 (0.659–12.566)	0.16

^a^Adjusted for Ki67, treatment arm, tumor grade, tumor size, ER status and node status

**Fig. 4:**
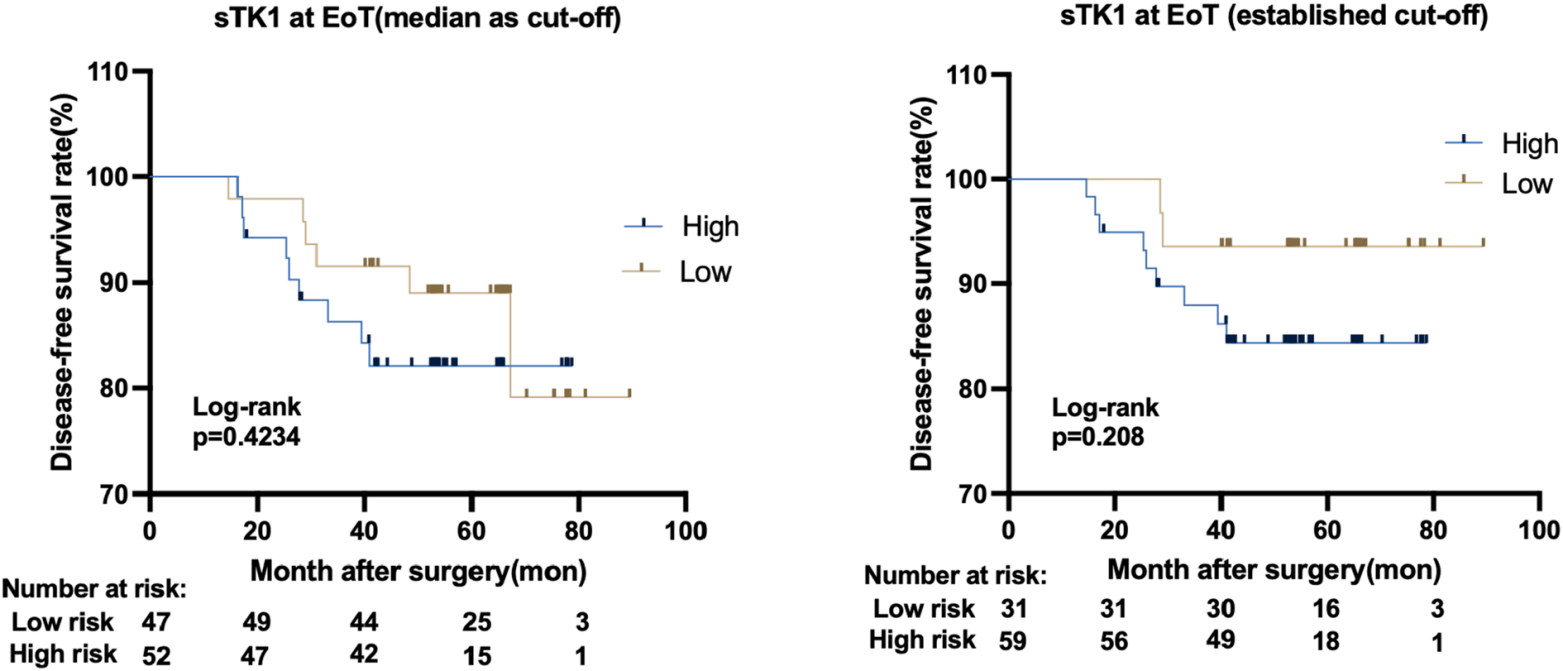
DFS probability according to sTK1 activity at visit EoT by **A** median value(422 DuA) as cut-off **B** 250 DuA as cut-off in a subset of non-pCR patients

A predefined cutoff of < 250 DuA is associated with a lower likelihood of disease progression in HR + HER2- Mbc [14] and was explored in the current study. Patients with low sTK1 at the end of adjuvant treatment (visit EoT) by both median and the predefined cutoff (250 DuA) had numerically better DFS (Supplementary Fig. 3), however the difference was not statistically significant in models adjusted for pCR, Ki67, treatment arm, tumor grade, tumor size, ER status, and node status (Supplementary Table 4). Subsequently, we assessed the prognostic value of sTK1 at visit EoT in a subset of non-pCR patients. A non-significant trend was observed among higher sTK1 activity at the end of treatment visit and worse survival outcomes (Fig. 4, Table 4).

**Table 4:** Association of sTK1 level at visit EoT with disease-free survival (DFS) in non-pCR patients

	Cox regression hazard ratio (95% confidence interval)			
	Unadjusted model		Adjusted model^a^	
	HR (95% CI)	*p* value	HR (95% CI)	*p* value
TK1 at EoT (high vs low by median cutoff)	1.521(0.5477–4.546)	0.4267	1.396 (0.4127 to 5.067)	0.5963
TK1 at EoT (high vs low by cutoff 250 DuA)	2.657(0.6842–17.44)	0.2115	2.952 (0.5277–24.83)	0.2542

^a^Adjusted for Ki67, treatment arm, tumor grade, tumor size, ER status and node status

## Discussion

One of the fundamental characteristics of cancer is its ability to proliferate, making cell proliferation a critical hallmark of the disease [1]. Changes in proliferation rates can serve as a significant indicator of tumor long-term prognosis and the response to early treatment. Liquid-based proliferation biomarkers have emerged as a promising non-invasive method for assessing these factors. The DiviTum® TKa assay platform has been validated for its reproducibility and has been found to perform favorably when compared to other assays[15]. In this prospective randomized trial, we evaluated the longitudinal serum TK1 activity and investigated its potential value in early HER2 + disease. Although clinical utility for this setting could not be demonstrated in our study, our major findings add information to current evidence of the sTK1 dynamics in BC and provide interesting insights to how sTK1 could be further investigated for various clinical uses.

Firstly, our study demonstrated dynamic sTK1 activity during different phases of HER2 + disease. We observed that the median sTK1 level of patients with detectable sTK1 at diagnosis was comparably higher than in a study on patients with clinical stage II HER2-negative breast cancer patients using the same assay [16], but lower compared to patients with advanced breast cancer [17], suggesting that sTK1 possibly reflects tumor burden, notwithstanding the difficulties of comparing different studies.

Furthermore, we observed an increase in sTK1 even after short exposure to neoadjuvant treatment, similar to our previous findings on HER2-negative breast cancer treated with chemotherapy [11], and in contrast with available data on neoadjuvant endocrine-treated disease [10]. This could be due to following explanations: (1) Effective targeted treatment induces cancer cell death, cytosolic TK1 is then released into the bloodstream; (2) Effective chemotherapy inhibits de novo dTMP synthesis pathway, and salvage pathway is effectively activated, leading to more TK1 uptake, thus more exocytosis/exosome TK1 is detected in the blood[18]. Therefore, sTK1 is more likely to be a metabolic marker as also indicated in previous clinical and preclinical studies [19, 20]. Interestingly, more patients had high sTK1 in the DPH arm than the TDM-1 arm during neoadjuvant treatment, probably indicating a larger metabolic change for patients receiving regimens containing traditional chemotherapeutics. For both treatment groups, sTK1 increased significantly from baseline to visit 2 in both pCR and non-pCR cases, we also observed a marginally significant change of sTK1 from visit 2 to visit 3 in non-pCR cases but not in pCR cases. However, neither baseline sTK1, sTK1 at cycle 2 or at cycle 4 by cutoffs at respective timepoints associated with pCR. Previous findings had linked lower sTK1 with a greater likelihood of treatment response of chemotherapy in lung cancer patients [18], which we did not observe with an important caveat however, the limited detection range of the assay that introduces informative missingness to the analyses. Similarly, an association between sTK1 levels at any timepoint during neoadjuvant therapy, or of sTK1 kinetics, with long-term survival was not observed. We have previously demonstrated that a greater early sTK1 increase during neoadjuvant therapy for HER2-negative breast cancer, mostly in highly proliferative tumors, and the extent of this early increase was associated with improved survival outcome. The reasons behind the lack of prognostic value for HER2-positive breast cancer in this study are unclear, whether it is due to small sample size with few events, the detection range of the assay, or the biology of the disease, so further investigation is warranted.

An intriguing finding of our study is the plausible prognostic value of sTK1 for patients with residual invasive HER2-positive breast cancer. Trastuzumab emtansine is the recommended post-neoadjuvant salvage therapy for such patients, even though three out of four patients treated with trastuzumab in the KATHERINE trial were disease-free at 3 years [21], patients that are currently overtreated with trastuzumab emtansine, with higher toxicity and increased cost as a result. Attempts to refine the post-neoadjuvant strategy by using the grade of histopathologic remission [22] or bespoke circulating tumor DNA panels [23] have shown clear clinical validity but have hitherto lacked clinical utility. Here, we show that sTK1 levels following surgery identify distinct prognostic groups within the population of patients with residual disease. Conceivably, by combining the well-validated Residual Cancer Burden index and sTK1, an assay of low complexity and cost, prognostication could be refined and patients with excellent prognosis be spared of unnecessary salvage treatment. Although our findings should be considered hypothesis generating due to their exploratory nature and few post-surgery relapses, the unmet clinical need to better stratify patients with residual invasive disease underscores the need for further validation of our observations in a larger cohort.

This is to the best of our knowledge the first study that longitudinally assessed serum TK1 levels for HER2 + BC patients in a prospective, randomized clinical trial with long-term follow-up of more than five years. Additionally, serum samples were collected at baseline and subsequent timepoints from most trial participants, ensuring adequate representation and minimizing informative missingness. On the other hand, our study has some limitations that need to be considered. Firstly, it is an exploratory biomarker study that relies on retrospective analysis of prospectively collected data and the findings lack validation. Secondly, the relatively small number of patients and survival events may have concealed associations with outcomes. Additionally, the TK1 assay itself has technical obstacles to overcome, such as a detection range adopted for HER2- negative disease, and currently no standard cutoffs for early-stage disease.

In conclusion, our study is, to the best of our knowledge, the first study that longitudinally assessed sTK1 as a putative long-term prognosticator in HER2 + breast cancer, both at baseline and following short-term exposure to neoadjuvant HER2-targeted therapy. While sTK1 levels and kinetics during treatment were generally not prognostic for short or long-term outcomes, the post-surgery prognostic value in patients that have not attained pCR warrants further investigation.

### Supplementary Information

Below is the link to the electronic supplementary material.

Supplementary file1 (PPTX 704 KB)

## Data Availability

The datasets analyzed during the current study are available from the corresponding author upon reasonable request.
