# Supplementary material for: The role of serum thymidine kinase 1 activity in neoadjuvant-treated HER2-positive breast cancer: biomarker analysis from the Swedish phase II randomized PREDIX HER2 trial
Source: Breast Cancer Res Treat. 2024 Jan 4;204(2):299–308. doi: 10.1007/s10549-023-07200-x (PMC10948570; doi:10.1007/s10549-023-07200-x)
Supplement: Supplementary file 1 — Supplementary file1 (PPTX 704 KB) [file 10549_2023_7200_MOESM1_ESM.pptx]

## Slide 1
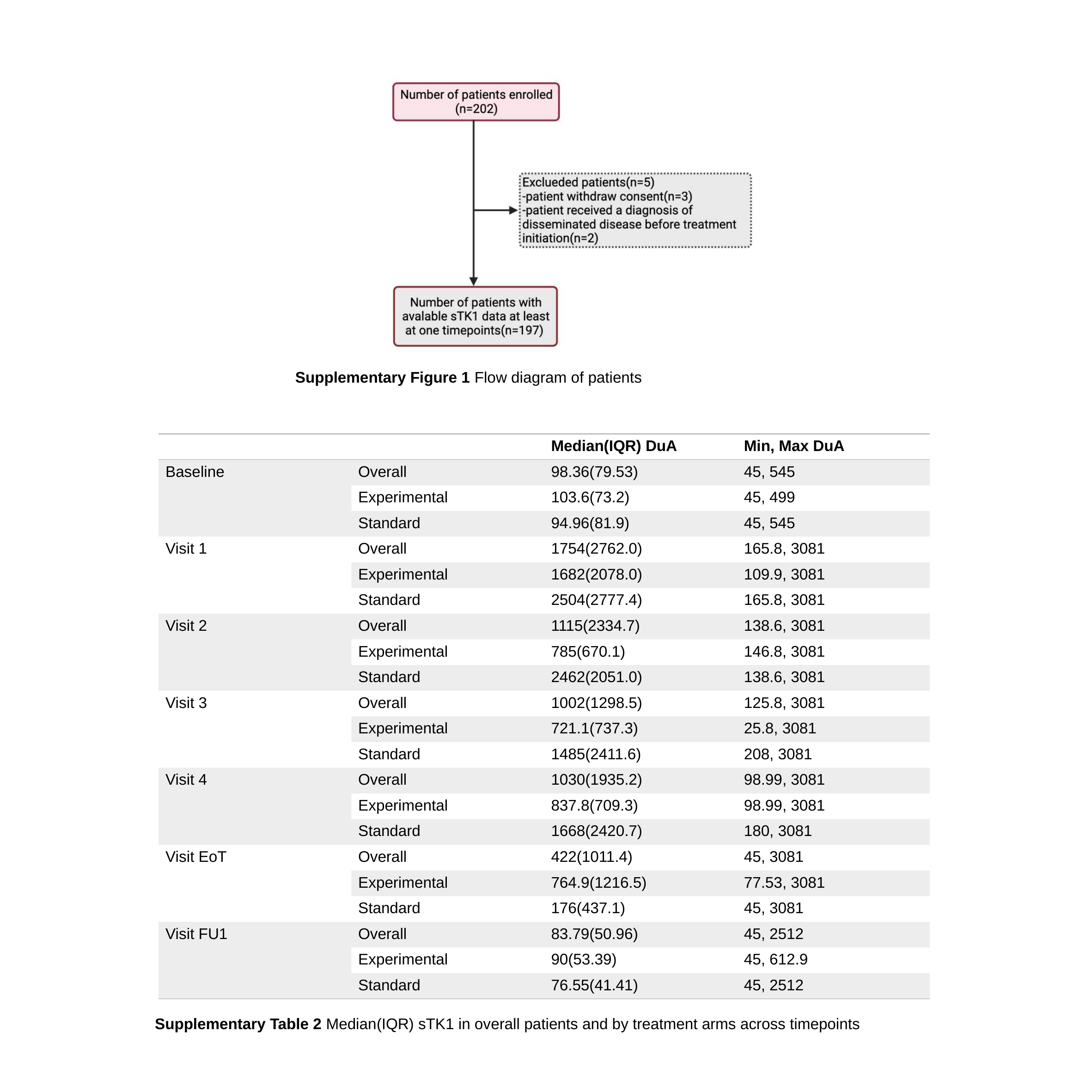

Supplementary Figure 1 Flow diagram of patients
| | | Median(IQR) DuA | Min, Max DuA |
| --- | --- | --- | --- |
| Baseline | Overall | 98.36(79.53) | 45, 545 |
| | Experimental | 103.6(73.2) | 45, 499 |
| | Standard | 94.96(81.9) | 45, 545 |
| Visit 1 | Overall | 1754(2762.0) | 165.8, 3081 |
| | Experimental | 1682(2078.0) | 109.9, 3081 |
| | Standard | 2504(2777.4) | 165.8, 3081 |
| Visit 2 | Overall | 1115(2334.7) | 138.6, 3081 |
| | Experimental | 785(670.1) | 146.8, 3081 |
| | Standard | 2462(2051.0) | 138.6, 3081 |
| Visit 3 | Overall | 1002(1298.5) | 125.8, 3081 |
| | Experimental | 721.1(737.3) | 25.8, 3081 |
| | Standard | 1485(2411.6) | 208, 3081 |
| Visit 4 | Overall | 1030(1935.2) | 98.99, 3081 |
| | Experimental | 837.8(709.3) | 98.99, 3081 |
| | Standard | 1668(2420.7) | 180, 3081 |
| Visit EoT | Overall | 422(1011.4) | 45, 3081 |
| | Experimental | 764.9(1216.5) | 77.53, 3081 |
| | Standard | 176(437.1) | 45, 3081 |
| Visit FU1 | Overall | 83.79(50.96) | 45, 2512 |
| | Experimental | 90(53.39) | 45, 612.9 |
| | Standard | 76.55(41.41) | 45, 2512 |
Supplementary Table 2 Median(IQR) sTK1 in overall patients and by treatment arms across timepoints

## Slide 2
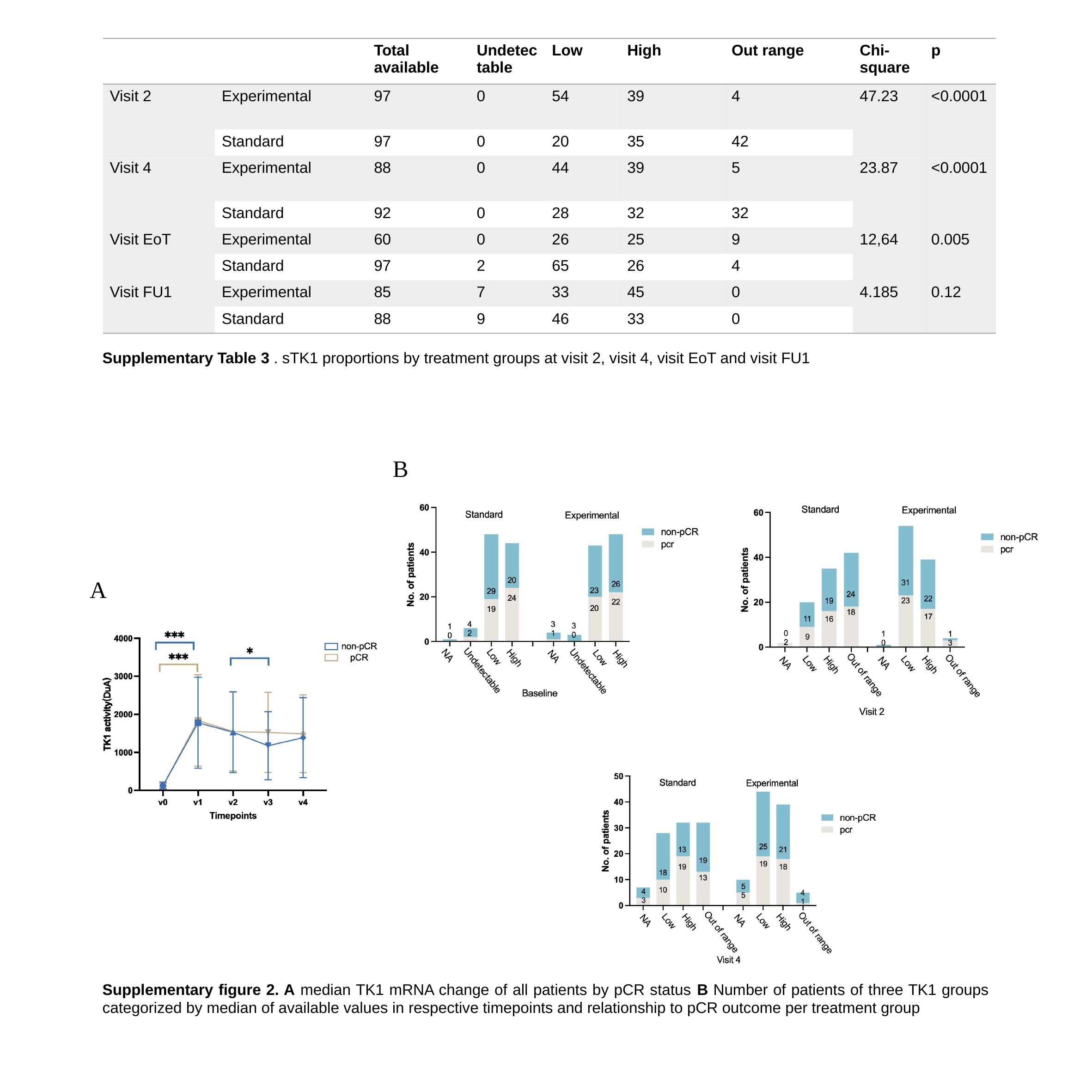

| | | Total available | Undetectable | Low | High | Out range | Chi-square | p |
| --- | --- | --- | --- | --- | --- | --- | --- | --- |
| Visit 2 | Experimental | 97 | 0 | 54 | 39 | 4 | 47.23 | <0.0001 |
| | Standard | 97 | 0 | 20 | 35 | 42 | | |
| Visit 4 | Experimental | 88 | 0 | 44 | 39 | 5 | 23.87 | <0.0001 |
| | Standard | 92 | 0 | 28 | 32 | 32 | | |
| Visit EoT | Experimental | 60 | 0 | 26 | 25 | 9 | 12,64 | 0.005 |
| | Standard | 97 | 2 | 65 | 26 | 4 | | |
| Visit FU1 | Experimental | 85 | 7 | 33 | 45 | 0 | 4.185 | 0.12 |
| | Standard | 88 | 9 | 46 | 33 | 0 | | |
Supplementary Table 3 . sTK1 proportions by treatment groups at visit 2, visit 4, visit EoT and visit FU1
B
A
Supplementary figure 2. A median TK1 mRNA change of all patients by pCR status B Number of patients of three TK1 groups categorized by median of available values in respective timepoints and relationship to pCR outcome per treatment group

## Slide 3
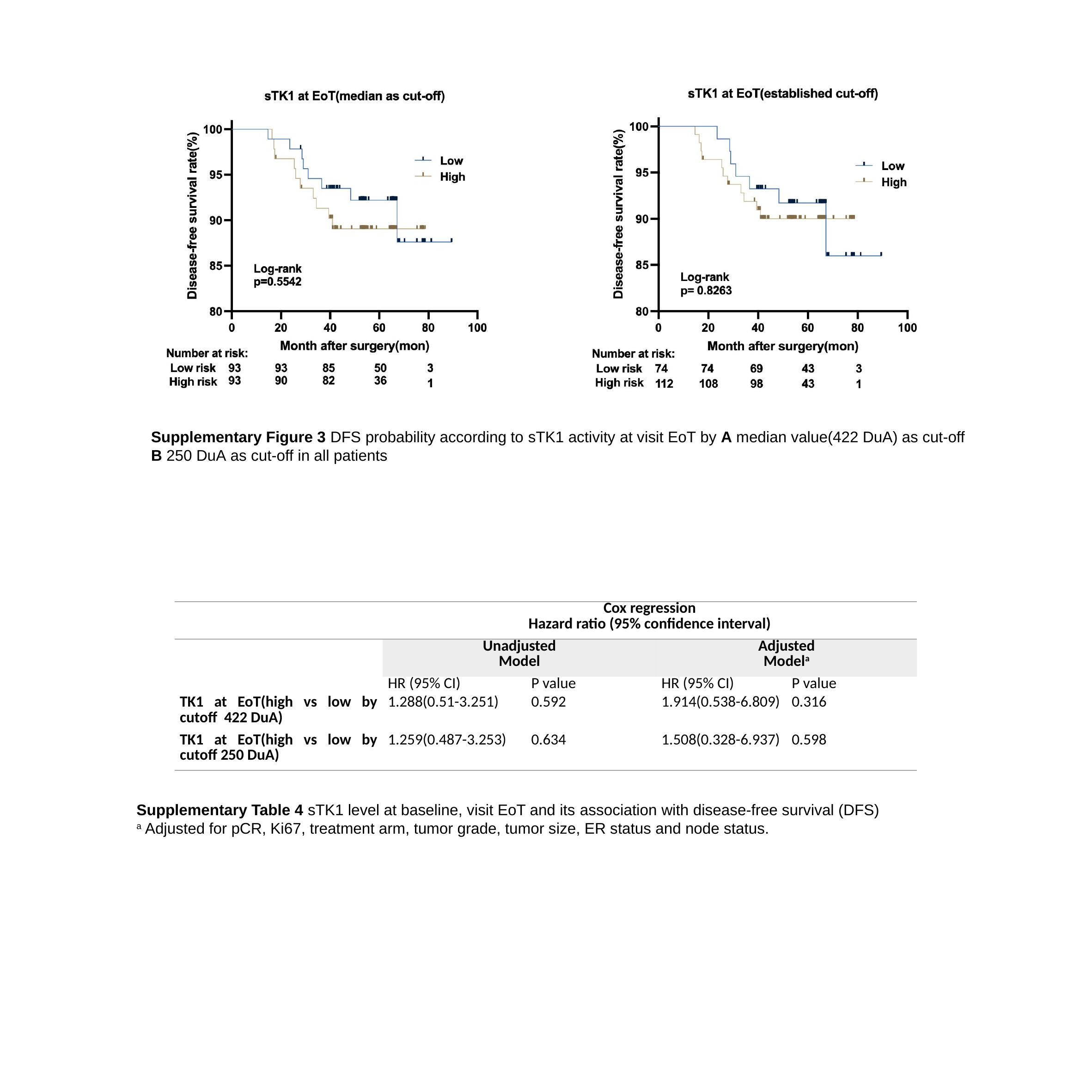

Supplementary Figure 3 DFS probability according to sTK1 activity at visit EoT by A median value(422 DuA) as cut-off B 250 DuA as cut-off in all patients
| | Cox regression Hazard ratio (95% confidence interval) | | | |
| --- | --- | --- | --- | --- |
| | Unadjusted Model | | Adjusted Modela | |
| | HR (95% CI) | P value | HR (95% CI) | P value |
| TK1 at EoT(high vs low by cutoff 422 DuA) | 1.288(0.51-3.251) | 0.592 | 1.914(0.538-6.809) | 0.316 |
| TK1 at EoT(high vs low by cutoff 250 DuA) | 1.259(0.487-3.253) | 0.634 | 1.508(0.328-6.937) | 0.598 |
Supplementary Table 4 sTK1 level at baseline, visit EoT and its association with disease-free survival (DFS)
a Adjusted for pCR, Ki67, treatment arm, tumor grade, tumor size, ER status and node status.
